# Barriers to Gait Training among Stroke Survivors: An Integrative Review

**DOI:** 10.3390/jfmk7040085

**Published:** 2022-10-13

**Authors:** Eveline Tavares, Joana Coelho, Patrícia Rogado, Rita Correia, Cidália Castro, Júlio Belo Fernandes

**Affiliations:** 1 Escola Superior de Saúde Egas Moniz, Caparica, 2829-511 Almada, Portugal; 2 Centro de Investigação Interdisciplinar Egas Moniz (CiiEM), 2829-511 Almada, Portugal; 3 Grupo de Patologia Médica, Nutrição e Exercício Clínico (PaMNEC), 2829-511 Almada, Portugal

**Keywords:** stroke, gait training, rehabilitation, barriers, exercise

## Abstract

Gait recovery is vital for stroke survivors’ ability to perform their activities associated with daily living. Consequently, a gait impairment is a significant target for stroke survivors’ physical rehabilitation. This review aims to identify barriers to gait training among stroke survivors. An integrative review was conducted following Whittemore and Knafl’s methodology. The research was carried out on the electronic databases Scopus, PubMed, and B-on, applying a time span of 2006 to 2022. A total of 4189 articles were initially identified. After selecting and analyzing the articles, twelve studies were included in the sample. This review allowed for the identification of several barriers to gait training among stroke survivors, which can be grouped into three categories: individual, environmental, and rehabilitation workforce-related barriers. These findings highlight that participation in gait training is not solely dependent on the stroke survivor. Instead, the uptake of rehabilitation programs may also depend on environmental and rehabilitation workforce-related factors.

## 1. Introduction

A stroke is a medical emergency that occurs when an artery to the brain becomes blocked or bursts, often leading to lasting brain damage, long-term disability, or even death [1]. 

Stroke is the second leading cause of death worldwide [2] and a major cause of adult neurological long-term disability [3], posing a staggering burden at both the individual and community levels. Furthermore, people who have had a stroke are at risk for recurrent strokes [3,4,5] and death [4,5]. 

A stroke is a high-volume medical condition. Globally, there are 12.3 million new strokes per year, with a prevalence of 101 million [2]. Furthermore, this prevalence is expected to increase in the upcoming years due to population aging [6,7]. 

With the numerous current advances in stroke-related clinical care, there have been significant improvements in survival rates post-stroke [2,3]. Therefore, suitable services are needed to provide care for stroke survivors in the community [8,9,10].

Stroke survivors should participate in regular rehabilitation programs to minimize disability, improve their overall fitness, and decrease the risk of recurrent strokes [8,9,10].

The long-term effects of a stroke depend on which part of the brain was affected and to what extent [1]. The most common types of disabilities after a stroke are impaired speech, physical limitations, and a gait disorder [1]. 

Evidence shows that over a quarter of stroke survivors are considered non-community walkers [11], and almost two-thirds report ongoing issues with mobility between one and five years after a stroke [12].

Gait impairments impact the performance of several activities of daily living according to the International Classification of Functioning, Disability and Health (ICF) activity and participation domains (e.g., mobility, self-care, domestic life, major life areas, and community, social, and civic life) [13]. 

In stroke survivors, walking speed is linked with functional ability and is associated with the hospital discharge location, future health status, and mortality [14]. Therefore, a gait disorder is a major target for stroke survivors’ physical rehabilitation [15,16].

As a result of technological development and improvements in health care, the worldwide life expectancy has increased by more than six years between 2000 and 2019 [17]. On the other hand, the aging population tends to have a higher prevalence of chronic diseases globally. Consequently, health care institutions have called for a change to achieve higher quality and efficiency in treating conditions. Nevertheless, several such initiatives have not succeeded [18]. Furthermore, the care practices should be based on evidence-based practice, meaning that the health care professionals quickly incorporate the best available research, along with their own experience and the patient’s situation, experiences, and desires, when deciding on treatment and efforts [19]. Although nowadays health care professionals resort to evidence-based practices, patients do not always receive the most effective treatments due to several barriers [20,21,22,23,24]. 

Studies involving stroke survivors have expressed the benefits of physical rehabilitation but have also identified barriers to participation in those exercise programs [25,26]. 

Enabling engagement in physical rehabilitation programs is the subject of a large body of research. A growing and significant field of research includes identifying “high level” factors that increase participation in rehabilitation programs at both the personal and environmental/facility levels [25,26]. 

The perceived barriers to any health behavior or healthcare intervention can significantly impact the likelihood of an individual’s acceptance of that particular behavior [22,27,28].

A randomized controlled clinical trial exploring the effects of continuous encouragement and verbal instruction was not effective in increasing stroke survivors’ adherence to physical activity [29]. In addition, the recommendations established for the general population will likely be ineffective for stroke survivors, e.g., the perceived barriers to any health behavior or healthcare intervention might differ significantly from those perceived by the general population due to the several long-term effects of stroke [30].

Considering the barriers to physical rehabilitation, studies show that barriers are not only centered on the stroke survivors but also on the rehabilitation workforce, the care environment, and policymakers [23,31]. By identifying the factors that can act as barriers in handovers between stroke survivors, health care professionals, and organizations, coordination and knowledge exchange in different actions can be developed [22]. A proper understanding of the barriers to participation in gait-related rehabilitation programs is vital to improve guidance to stroke survivors during therapy and further develop effective physical therapy interventions [8,9,10]. Without understanding the barriers associated with participation in gait training among stroke survivors, developing effective programs with sustainable outcomes is difficult. 

This review aims to identify the barriers to gait training among stroke survivors.

## 2. Methods

### 2.1. Design

An integrative review was conducted based on the methodological approach proposed by Whittemore and Knafl [32]. This approach involves five stages: (1) problem identification, (2) literature search, (3) data evaluation, (4) data analysis, and (5) presentation.

In accordance with the proposed methodology, the following research question was generated in line with the population, intervention, and context (PCC) questions to answer the first stage: problem identification. What barriers prevent stroke survivors from participating in gait training?

### 2.2. Search Methods

The literature search was carried out to achieve an overview of this broad field of literature using Scopus, PubMed, and B-on’s databases. The final search was performed in August, 2022.

Both free text and MeSH health sciences descriptors were used on the individual databases combined with Boolean operators using the following search string: ((stroke) AND (barriers) OR (difficulties) AND (gait training) OR (rehabilitation)).

The selection criteria were documents written in Portuguese or English, published between 2006 and 2022, which addressed or referred to the barriers for stroke survivors to participate in gait training. All documents that did not meet the selection criteria were excluded from the review.

### 2.3. Study Selection

To increase consistency, two researchers carried out the search, selection, and extraction of data independently. After eliminating duplicates, researchers proceeded with a selection process that comprised three phases. In the first phase, researchers screened the titles, followed by the abstract analysis. Finally, researchers obtained the full text of relevant documents and read them thoroughly. This process allowed for verifying the relevance and appropriateness of the selected documents according to the inclusion and exclusion criteria and the research question. When it was not clear whether the article fitted this review, it was automatically moved to the next phase. In cases of disagreement, a third reviewer made the final decision.

### 2.4. Data Evaluation

Researchers evaluated the quality of the selected research studies using the Joanna Briggs Institute levels of evidence and grading, ranging from 3e to 4d. The Joanna Briggs Institute Critical Appraisal Checklist was used to appraise each study. The bias risk percentage calculation was performed according to the following procedure: (i) scores below 49% were considered to possess a high risk of bias, (ii) between 50% and 69% a moderate risk of bias, and (iii) more than 70% a low risk of bias.

The methodological rigor of the studies selected by researchers ranged from 72.7% to 100%, which was considered a low risk of bias.

### 2.5. Data Analysis

Data were extracted from primary sources with the help of a data extraction form. The extracted data included the study authors, publication year, title, design, aim, and findings. All data items extracted were cross-checked. 

Two researchers reviewed data independently and manually coded them using inductive analysis to identify and group common categories across the collected data.

## 3. Results

**Figure 1:**
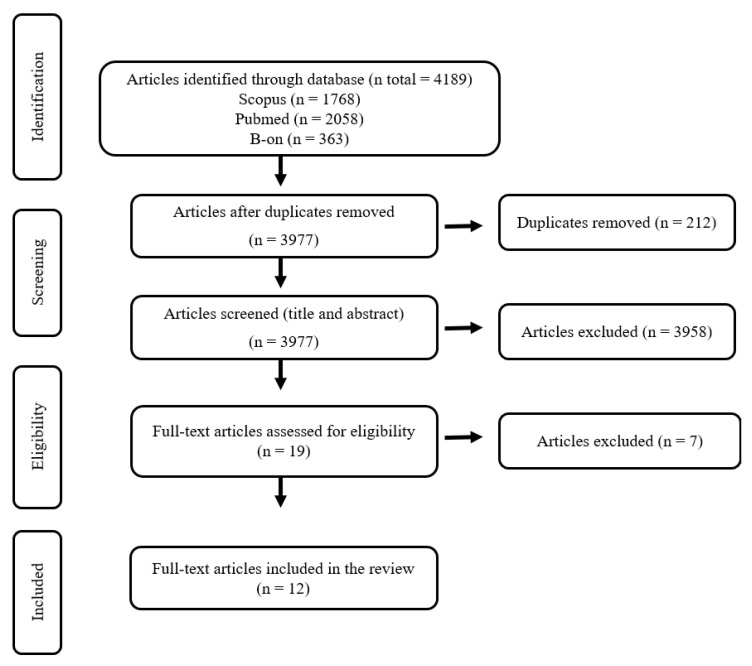
PRISMA flow diagram for study selection.

The initial database search identified 4189 articles. After the duplicate articles were removed, 3977 titles and abstracts were reviewed, of which 19 were considered suitable for a full-text review. At the end of the screening process, 12 studies met the eligibility criteria and were included in this review. The flow chart describing the screening process is presented in Figure 1.

This integrative literature review allowed for the identification of twelve articles that focus on the barriers for stroke survivors to participate in gait training. Out of the twelve studies, there were four studies conducted in the United States of America [25,31,33,34], two in Canada [26,35], two in the United Kingdom [36,37], one in Italy [38], one in Australia [39], one in Brazil [40], and another in the Netherlands [41]. 

A summary of the included articles with an overview of their key characteristics and findings is provided in Table 1.

The data analysis revealed several barriers to stroke survivors’ participation in gait training. Using an inductive analysis process, we grouped the different interventions into three categories based on the differences and similarities found between them. Each category is detailed below.

### 3.1. Category 1: Individual Barriers

This review identified individual barriers that focus on four main areas: physical, social, cognitive, psychological, and economic. 

Physical impairments due to stroke are a barrier to engaging in gait training. These impairments include vision problems [25,33,40,41], a lack of energy [25,31,37,40,41], pain [25,40,41], and motor impairments related to hemiplegia or spasticity [25,33,34,35,36,39,41].

The cognitive issues include recurrent episodes of distraction or a deficit of attention [25,41], diminished stimulus processing [25,41], disorientation [25,39], memory loss [25,39], and problem-solving difficulties [25,39].

At a psychological level, several studies identify the lack of motivation to perform any type of exercise [26,31,33,35,36,40], an alteration in patients’ mental health status due to feelings of depression [35], and even the fear of falling and losing one’s balance [26,34,37]. The studies also identified that some stroke survivors believe exercise will not improve their condition [31,40]. 

The social issues include insufficient time to attend training sessions due to difficulties in schedule management [25,31,34,35] and family obligations [35]. Several studies also identified that stroke survivors lack knowledge about what to do and how to access services [25,36,40].

One’s financial capacity can be a significant barrier to participating in gait training [25,31,36]. Even if the programs are offered for free, in some cases, the costs associated with travel in itself harm a patient’s family budget.

### 3.2. Category 2: Environmental Barriers

Several studies reported specific environmental barriers that interfered with or prevented stroke survivors’ participation in gait training, such as the absence of familial and social support [25,34,40] or the lack of awareness of opportunities [25,31,40]. In addition to the lack of awareness of opportunities, the studies also point out an access shortage, as stroke survivors highlighted a shortage of offered programs [40]. Hence, they were unable to engage in gait training. When patients do engage in training, issues with transportation [25,31,33,36,40], accessibility [25,40], and safety in the built environment [25] may also be barriers to participation.

### 3.3. Category 3: Rehabilitation Workforce Barriers

In addition to program access shortages, the studies identified that stroke survivors reported that the programs they attended lacked tailored interventions [26,31,33]. They felt that there was a lack of exercise options, and that training sessions could become boring or monotonous. Stroke survivors have different problems, so they should receive interventions personalized to their different skills and needs.

Other barriers to participation are focused on the rehabilitation workforce’s knowledge and skills. The studies identified that stroke survivors consider that the lack of support from qualified personnel [26], their educational level, and expertise affects the treatment delivered [38,39]; hence, this can act as a barrier to participation in gait training.

## 4. Discussion

The current review aims to provide a comprehensive understanding of the barriers faced by stroke survivors to participate in gait training. A total of three categories of barriers (individual, environmental, and rehabilitation workforce-related barriers) were identified from twelve studies.

This study yielded many significant findings. First, the results demonstrate that the barriers to participating in gait training are highly complex, encompassing several dimensions. 

Many stroke survivors must leave with physical and/or cognitive impairments [1,2,42,43]. The studies revealed that these impairments could limit the stroke survivors’ participation in rehabilitation programs due to the lack of skills or an inability to perform the exercise program [25,26,31,33,34,35,36,37,39,40,41]. Furthermore, this lack of skill or inability to perform exercise can lead to a lack of motivation [26,31,33,35,36,37,40], which will have a great impact on the patient’s adherence to the rehabilitation programs [44,45]. Suppose we add a potential rehabilitation workforce’s inability to provide personalized care to the patient’s different skills and needs. In that case, it is possible to perceive the complexity and interconnection between the various barriers. Therefore, health care professionals must consider the person’s skills, values, interests, personality type, and aptitudes to personalize the rehabilitation program and eliminate or minimize the effects of possible barriers [22,23]. 

A second significant finding of this review is that several studies identify an access shortage to rehabilitation programs [25,33,36,38,39]. In addition, when they engage in training programs, stroke survivors face issues regarding transportation, accessibility, and safety in the built environment [25,26,31,33,36,40].

Stroke survivors must have opportunities within their environment to attend gait training. In addition, there must be a diversity of offers so that the person can select a program that best suits their personality [8,9,10,15,16]. 

It is evident that the successful rehabilitation of stroke survivors requires an approach involving greater program offers. In the face of limited resources, stroke survivors will likely become unmotivated after realizing that the resources are scarce and scattered [46,47].

A person’s economic context should also be considered, as this barrier may have a crucial impact on participating in gait training [25,31,36,48]. For example, suppose stroke survivors do not have the financial capacity to support their daily expenses. In that case, it is more than reasonable to assume they will not contemplate engaging in other activities. However, considering the evidence gathered from different areas of rehabilitation care, there seems to be a consensus that the cost associated with intervention may not be perceived as a barrier as long as the cost is reasonable enough [49].

A third important finding is the significance of familial and social support for the participation of stroke survivors in gait training. If one’s family and significant others are not supportive of engaging in an active rehabilitation program, the patients are likelier to show a lower adherence to exercise [25,34,35,40].

Health care professionals must ensure that stroke survivors and family caregivers are engaged with a family-centered approach. Stroke survivors and family caregivers should be viewed as a unit. Therefore, the assessment of need should not be centered only on the patient but should also include the needs of the family [50,51]. 

Another barrier to participation in gait training involved the societal attitudes toward people with a disability [25,35]. As stroke impairments may be visible to others, they can lead to stigmatizing social experiences. Consequently, it is necessary to remove the stigma against disability so that stroke survivors can receive the help they need to promote their rehabilitation [52,53].

A fourth significant finding of this review focuses on the importance of providing tailored interventions to improving stroke survivors’ participation in gait training [26,38,39].

It is well known that treatment strategies tailored to an individual lead to better clinical outcomes and higher levels of adherence and satisfaction [54,55,56]. Unfortunately, there are no strategies that have universal applicability [57]. Health care professionals must be able to personalize care interventions to each patient’s needs. Personalized care can be a key to increasing the likelihood of keeping gait rehabilitation programs challenging and engaging for stroke survivors [58]. However, adjusting exercises to each patient’s physical and cognitive abilities can be challenging [24]. Therefore, health care professionals need the knowledge and skills to adapt the training to each person [23].

### Limitations

The findings of this review revealed that there are several significant barriers for stroke survivors to participate in gait training. A key strength of this review lies in the fact that several studies reported similar data, which allows us to be confident in the identified results. However, the review is limited in several ways. First, as there are no specific MeSH health sciences descriptors for barriers, we relied on free text. Second, the databases’ restrictions and imposed time limits may influence the results obtained. Third, we researched the literature that was written in only the English and Portuguese languages, which may have excluded potentially relevant articles.

This review has revealed that data regarding the perceived barriers to participating in a gait rehabilitation program among stroke survivors are emerging. However, the geographical distribution of the selected articles is limited to the United States of America, Canada, the United Kingdom, Italy, Brazil, Australia, and the Netherlands. Therefore, further research is needed, particularly in countries outside of North America, as stroke survivors in different countries may diverge in their perceptions of engaging in gait rehabilitation programs.

## 5. Conclusions

This review allowed for the identification of several barriers to stroke survivors’ participation in a gait rehabilitation program, the bulk of which can be grouped into three categories: individual, environmental, and rehabilitation workforce-related barriers. 

These findings highlight that participation in gait training does not solely depend on the stroke survivor. Instead, the uptake of rehabilitation programs might rely on the assistance and support received from family caregivers and health care professionals.

This review can contribute to improving care for stroke survivors. Health care professionals must be aware of the barriers to participating in a gait rehabilitation program among stroke survivors. Understanding these barriers will enable the rehabilitation workforce to tailor interventions to target barriers and provide more focused support and guidance to stroke survivors.

## Figures and Tables

**Table 1:** Data extraction and synthesis.

Author/Year/Title/Country	Study Design/Aim	Categories and Subcategories of Barriers Identified per Study
Hammel, Jones, Gossett, and Morgan [25] (2006) Examining barriers and supports to community living and participation after a stroke from a participatory action research approach United States of America	Qualitative descriptive study To identify barriers to full participation and exmanie action-planning strategies to address these barriers within community settings and businesses and to implement policy and system changes to effect participation opportunities at the broader societal level.	Individual barriers: ○ Awareness of opportunities to participate ○ Actively problem solves and strategizes ○ Plans ahead for future participation ○ Seeks information and assistance as needed ○ Strategizes navigation and orientation ○ Health status: physical/energy/pain/mental health/cognitive issues ○ Sensory status: vision/hearing ○ Communication status ○ Activity: bathroom use/community mobility/schedule management Environmental level ○ Physical: access and safety in the built environment/access to and use of assistive technology/bathrooms’ accessibility and use ○ Social: family and friends support engagement in participation ○ Social: peers support participation/staff support participation/societal attitudes and beliefs ○ Transportation: access/availability/quality and reliability/affordability ○ Resources: access to training and education/opportunities to practice and integrate activities into the routine/information access/access to peer mentoring ○ System promotes a very unrestrictive environment ○ Economic resources are available to support participation ○ National policy: accessibility of buildings facilities/accessible transportation/accessible parking
Damush, Plue, Bakas, Schmid, and Williams [33] (2007) Barriers and facilitators to exercise among stroke survivors United States of America	Qualitative descriptive study To elicit barriers to and facilitators of exercise after stroke.	Perceived stroke impairments discourage activity en-gagement ○ Physical limitation ○ Impaired vision ○ oWalking difficulties Lack of motivation Environmental factors ○ Lack of exercise options ○ Lack of training programs ○ Lack of transportation
Rimmer, Wang, and Smith [31] (2008) Barriers associated with exercise and community access for individuals with stroke United States of America	Cross-sectional study To examine the multidimensional nature of barriers to physical activity reported by people who suffered stroke.	Environmental/Facility ○ Cost of the program ○ Lack of transportation ○ Unaware of a fitness center in the area ○ Do not feel the trainer in facility is able to help ○ Not comfortable exercising in a facility Personal ○ Do not know how to exercise ○ Do not know where to exercise ○ Social: peers support participation/staff support participation/societal attitudes and beliefs ○ Lack of energy and motivation ○ Exercise will not improve my condition ○ I am too lazy to exercise ○ Health concerns prevent me from exercising ○ Exercise is too difficult/boring or monotonous ○ Lack of time ○ Exercise will make my condition worse
Zalewski and Dvorak [34] (2011) Barriers to physical activity between adults with stroke and their care partners United States of America	Cross-sectional study To describe the daily physical activity patterns and perceived barriers to increasing physical activity for adults who have completed their rehabilitation after stroke and for their primary care partners.	Lack of willpower Lack of skill Fear of Injury Lack of Energy Lack of Resources Social influence Time
Jurkiewicz, Marzolini, and Oh [35] (2011) Adherence to a home-based exercise program for individuals after stroke Canada	Cross-sectional study To retrospectively identify factors that affect adherence to a home-based exercise program adapted for stroke patients.	Lack of motivation Musculoskeletal issues Too fatigued Not enough time Family obligations Depression Not enjoyable No benefits Too difficult
Simpson, Eng, and Tawashy [26] (2011) Exercise perceptions among people with stroke: Barriers and facilitators to participation Canada	Qualitative descriptive study To explore the perceptions of exercise among stroke survivors, including their concepts and definitions of exercise, as well as their perceptions of barriers and facilitators to exercise.	Lack of balance Fear of falling and losing one’s balance Ability and inability to perform exercise activities Lack of support from qualified personnel Lack of motivation
Nicholson, Sniehotta, van Wijck, Greig, Johnston, McMurdo, Dennis, and Mead [36] (2013) A systematic review of perceived barriers and motivators to physical activity after stroke United Kingdom	Systematic review To systematically review the literature to identify all studies examining perceived barriers and motivators to physical activity after stroke.	Personal barriers ○ Lack of motivation ○ Physical difficulties ○ Lack of knowledge about what to do and how to access services. Environmental barriers ○ Physical and transportation access to services ○ Economic costs
Nicholson, Greig, Sniehotta, Johnston, Lewis, McMurdo, Johnston, Scopes, and Mead (2017) [37] Quantitative data analysis of perceived barriers and motivators to physical activity in stroke survivors United Kingdom	Cross-sectional study To explore stroke survivors’ perceived barriers, motivators, self-efficacy, and intention to undertake physical activity	Poor health Fatigue Fear of falling or damage health
Débora Pacheco, Guimarães Caetano, Amorim Samora, Sant’Ana, Fuscaldi Teixeira-Salmela, and Scianni [40] (2021) Perceived barriers to exercise reported by individuals that suffered stroke and who are able to walk in the community. Brazil	Cross-sectional study To identify the perceived barriers to exercise, which could be modified, as well as the associated factors in people at the sub-acute post-stroke stages, who were able to walk in the community.	Fatigue after exercising Availability and distance from the exercise places Lack of a person to help Knowledge on how to practice exercise Lack of energy Lack of transportation and accessibility Lack of knowledge on where to practice exercises Lack of interest Pain Belief that exercise is boring and unnecessary Visual and balance problems
Goffredo, Infarinato, Pournajaf, Romano, Ottaviani, Pellicciari, Galafate, Gabbani, Gison, and Franceschini [38] (2020) Barriers to sEMG Assessment During Overground Robot-Assisted Gait Training in Subacute Stroke Patients Italy	Cross-sectional study To assess the barriers to the implementation of a ElectroMyoGraphy-based assessment protocol in a clinical context for evaluating the effects of Robot-Assisted Gait Training in subacute stroke patients.	Educational level Expertise of the members of staff
Tole, Raymond, Williams, Clark, and Holland [39] (2020) Strength training to improve walking after stroke: how physiotherapist, patient and workplace factors influence exercise prescription Australia	Qualitative descriptive study To explore perceived barriers and facilitators that influence Australian physiotherapeutic practices when prescribing strength training to stroke survivors undergoing gait rehabilitation.	Patient factors influence the approach to training ○ Therapy programs maximize patient engagement ○ Degree of physical and cognitive impairment directs therapy ○ Presence of co-morbidities affected therapist’s confi-dence in the implementation of training programs Interpretation and implementation of strength-training principles is diverse ○ Depth of knowledge of the principles of strength training varied ○ Movement quality is prioritized ○ Principles of strength training are inconsistently ap-plied ○ Therapists prioritize repetition and prescription of functional tasks ○ Therapist’s preference determines the choice of ap-proach ○ Research engagement influences the delivery of strength training Workplace context affects the treatment delivered ○ Resource limitations ○ Clinical preference of colleagues influences practice
de Rooij, van de Port, van der Heijden, Meijer, and Visser-Meily [41] (2021) Perceived barriers and facilitators for gait-related participation in people after stroke: From a patients’ perspective Netherlands	Qualitative descriptive study To explore barriers and facilitators for gait-related participation from the perspective of people who suffered stroke.	Neuromusculoskeletal and movement-related functions ○ Motor impairment ○ Decreased muscle strength ○ Decreased endurance ○ Decreased coordination ○ Fatigue Sensory functions and pain ○ Impaired visual function ○ Dizziness, tingling, or unusual sensations ○ Pain Mental functions. ○ Motor dual tasks ○ Allocation of attention ○ Diminished stimulus processing ○ Delayed information processing

## Data Availability

The data presented in this study are available on request from the author, J.B.F.
